# The Utilization and Choices of Aneuploidy Screening in a Midwestern Population

**DOI:** 10.1007/s10897-014-9711-x

**Published:** 2014-10-01

**Authors:** Jeffrey M. Dicke, Lindsey Van Duyne, Rachael Bradshaw

**Affiliations:** 1 Department Obstetrics and Gynecology, Washington University in St. Louis, School of Medicine, 660 S. Euclid Avenue, Campus Box 8064, St. Louis, MO 63110-1094 USA; 2 Westminster College, Fulton, MO USA; 3 Department of Pediatrics, Saint Louis University School of Medicine, St. Louis, MO USA

**Keywords:** Aneuploidy, Screening, First trimester, Second trimester, Amniocentesis, CVS

## Abstract

The types, interpretation, and use of first- and second-trimester aneuploidy screening are often unclear for many women. This impairs appropriate decision making and understanding of the implications of prenatal genetic testing options. The purpose of this study was to examine the utilization of Stepwise Sequential screening in our Midwestern population, demographic factors associated with choice of screening and method of risk reporting and it’s potential impact on women’s choices. First trimester screening was performed for 2,634 women during the study period. Results were not reported or “framed” as “positive” or “negative”. Rather, the specific age-risk and screen-risk for T21 were relayed, along with options for follow-up Stepwise Sequential screening and invasive testing. Nearly 80 % of women declined Stepwise Sequential screening. Minorities and women of lower education were least likely to pursue further screening. Less than 4 % of the study population elected invasive testing. First trimester screening was associated with a 53 % reduction in amniocenteses and 20 % fewer CVS’s compared to pre-first trimester screening availability. Reporting age-and screen-risks for T21, rather than classifying results as “positive” or “negative” based on a pre-determined threshold, was associated with a low uptake of further testing.

## Introduction

In January 2007, the American College of Obstetricians and Gynecologists (ACOG) Practice Bulletin Number 77 recommended “screening and invasive diagnostic testing should be available to all women who present for prenatal care before 20 weeks of gestation regardless of maternal age”. Several strategies for screening have been proposed based on the first trimester combined test (nuchal translucency (NT) measurement and biochemical markers) and second trimester maternal serum quadruple (Quad) screen. The highest detection rates and lowest screen positive rates have been reported for Integrated screening. This includes first trimester nuchal translucency, pregnancy-associated plasma protein A (PAPP-A) and human chorionic gonadotropin (hCG) plus maternal serum Quad screening in the mid-trimester, with the results provided only after all tests are completed. Patient anxiety during the delay between presentation for screening and results make this an unacceptable option for many patients. Proposed alternative screening options include Stepwise Sequential screening and Contingent Sequential screening based on classifying women as high- or low-risk using a predetermined threshold (American College of Obstetricians and Gynecologists ACOG 2007). Stepwise Sequential screening is first trimester combined testing plus maternal serum Quad screening with results provided after each test. Contingent Sequential screening is first trimester combined testing with only women having a risk between 1:30 and 1:1,500 for trisomy 21 (T21) proceeding to maternal serum Quad screening.

The manner in which risks are communicated can influence how this information is utilized. Prenatal aneuploidy screening results are often reported or “framed” as “positive” or “negative” based on a defined threshold. This is usually the risk of a 35-year old woman having a child with T21 or trisomy 18 (T18). However, there is little information about the effects of using verbal descriptions of risk versus numerical data. In a survey of 169 British antenatal clinics about the method of communicating Down syndrome screen negative results, 44 % used a verbal phrase (low risk, screen negative, within normal limits, not needing further action, among others), 16 % gave a numeric risk figure and 40 % used both a verbal phrase and numeric risk (Marteau 1999). The value of these different techniques for discussing risks is uncertain.

The goals of this study were to examine: 1- the uptake of Stepwise Sequential screening in our primarily urban, Midwestern population, 2- the potential affects of ethnicity, education and proximity to the medical center on willingness to proceed with further testing, and 3- our method of risk reporting, which emphasized a woman’s age-risk for T21 and adjusted risk after screening, rather than classifying a result as “positive” or “negative”, or high-risk, or low-risk.

## Methods

This is a retrospective cohort study of all patients who completed first trimester NT and biochemical screening at our institution between June 2007 and February 2010. This study was approved by the Washington University Human Research Protection Office, which waived informed consent. Inclusion criteria included women with singleton gestations who completed combined first trimester screening with NT and the serum markers PAPP-A and hCG. For the first 3 months of the study period, the laboratory utilized at this institution used total beta-hCG. Thereafter, free beta-hCG was the analyte employed. Exclusion criteria included multiple gestations, blood testing not performed or analyzed and inability to obtain a NT measurement. All NT measurements were performed by physicians or sonographers certified in NT measurement by either the Fetal Medicine Foundation or the Nuchal Translucency Quality Review (NTQR) program.

Patient’s were categorized at the time of their first trimester screening visit into one of three groups as follows: U = undecided as to whether they wished to proceed with Stepwise Sequential screening; Y = yes, they planned to pursue Stepwise Sequential screening; or N = no, they do not plan to have Stepwise Sequential screening. Results of first trimester screening were not reported as positive or negative. The phone call and/or letter informing patients of their first trimester screen results indicated the patient’s age-risk for (T21) and trisomy 18 (T18) and their revised risks for same based on the NT and biochemical markers. The determination of whether a patient was of high enough risk to warrant further screening or testing was at the patient’s discretion. All phone calls and/or result letters also again reviewed the patient’s subsequent options, including no further screening, maternal serum Quad screening, mid-trimester ultrasound and invasive testing via chorionic villus sampling (CVS) or amniocentesis. The patient’s decision about whether to proceed with Stepwise Sequential screening (both before and after their first trimester screening results) were recorded in a computerized database for subsequent correlation with variables including ethnicity, level of education, distance from our medical center, NT measurement, crown rump length, age- and screen-risks for T21, results of invasive testing and pregnancy outcomes.

In order to assess the impact of non-invasive first trimester screening and Stepwise Sequential screening (first trimester screening plus maternal serum Quad screening) on the utilization of amniocentesis and CVS, the number of these procedures was compared as follows: for a 2-year period prior to the initiation of first trimester screening (January 2000–December 2001), for a 2-year period when first trimester screening was utilized but prior to the availability of Stepwise Sequential screening (June 2005–May 2007) and for the 2-year period following initiation of Stepwise Sequential screening (July 2007–June 2009).

Relationship between decision status (Yes, No, Undecided) and various characteristics were compared using the chi-square test or Fisher’s exact test, as appropriate. Differences in the counts of invasive procedures performed across time periods were evaluated using simple Poisson regression models, where time period (2000–2001, 2005–2007, and 2007–2009) was included using indicator variables.

## Results

First trimester only screening was available at our institution beginning in 2003. The option of Stepwise Sequential screening began to be routinely offered in June 2007. The study population consists of 2,643 patients who met inclusion criteria during the study period (June 2007–February 2010). During this time, an acceptable NT measurement was unable to be obtained in 8 patients (0.3 % of the initial study population). At the time of the first trimester examination, 1,926 (72.8 %) were undecided regarding Stepwise Sequential screening (U group) pending their first trimester results. 434 patients (16.4 %) indicated they planned to return for Stepwise Sequential screening, regardless of their results (Y group) and 283 patients (10.7 %) declined Stepwise Sequential screening at the time of their first trimester screening (N group).

The numbers of patients who actually pursued Stepwise Sequential screening are listed in Table 1. This included a minority of the Undecided and No groups (14.9 % and 1.4 %, respectively) and only 56.4 % of those who originally intended to have Stepwise Sequential screening. Overall, 79.8 % of the study population elected not to pursue follow-up screening.

**Table 1:** Stepwise sequential screening following first trimester combined screen

Group	N	No SS screening	Follow-up SS screening	*P*
Undecided	1,926 (72.9 %)	1,639 (85.1 %)	287 (14.9 %)	<0.0001
Yes	434 (16.4 %)	190 (43.8 %)	244 (56.2 %)	
No	283 (10.7 %)	279 (98.6 %)	4 (1.4 %)	
	2,643 (100 %)	2,108 (79.8 %)	535 (20.2 %)	
			U vs Y	<0.0001
			U vs N	<0.0001
			Y vs N	<0.0001

The relation between education level and choice of screening is presented in Table 2. In the group of women who did not plan to pursue Stepwise Sequential screening there were a significantly greater number who were not high school graduates and significantly fewer who were college graduates compared to women in the other two groups. Table 3 demonstrates that women who declined Stepwise Sequential screening were more likely to be Black and Hispanic and less likely to be White, than women in the other groups. Table 4 indicates that distance from the medical center did not correlate with the decision to pursue or decline Stepwise Sequential screening.

**Table 2:** Eduction level and decision to pursue stepwise sequential screening

Group	N	Some high school	HS grad some college	College grad post grad	*P* <0.0001
Undecided	1,923	68 (3.5 %)	501 (26.1 %)	1,354 (70.4 %)	
Yes	434	11 (2.5 %)	115 (26.5 %)	308 (71.0 %)	
No	282	22 (7.8 %)	111 (39.4 %)	149 (52.8 %)	
∑	2,639	101 (3.8 %)	727 (27.5 %)	1,811 (68.6 %)	
			U vs. Y	0.58	
			U vs. N	<0.0001	
			Y vs N	<0.0001

**Table 3:** Ethnicity and decision to pursue stepwise sequential screening

Ethnicity							
Group	N	White	Black	Asian	Hisp	Other	*P* <0.0001
Undecided	1,926	1,395 (72.4 %)	293 (15.2 %)	106 (5.5 %)	49 (2.5 %)	83 (4.3 %)	
Yes	434	326 (75.1 %)	50 (11.5 %)	18 (4.1 %)	8 (1.8 %)	32 (7.4 %)	
No	283	165 (58.3 %)	71 (25.1 %)	10 (3.5 %)	17 (6.0 %)	20 (7.1 %)	
∑	2,643	1,886 (71.4 %)	414 (15.7 %)	134 (5.1 %)	74 (2.8 %)	135 (5.1 %)	
					U vs. N	0.01	
					U vs. N	<0.0001	
					Y vs. N	<0.001

**Table 4:** Distance from medical center and decision to pursue stepwise sequential screening

			Miles		*P*
Group	N	0–20	21–50	>50	0.14
Undecided	1,926	1,293 (67.1 %)	507 (26.3 %)	126 (6.5 %)	
Yes	434	284 (65.4 %)	114 (26.4 %)	36 (8.3 %)	
No	283	170 (60.1 %)	88 (31.1 %)	25 (8.8 %)	
∑	2,643	1,747 (66.1 %)	709 (26.8 %)	187 (7.1 %)	
				U vs. Y	0.42
				U vs. N	0.05
				Y vs. N	0.32

Tables 5 and 6 demonstrate that the percentages of women at risk for T21 were similar between the groups.

**Table 5:** Age risk for T21 and decision to pursue stepwise sequential screening

Group	≥1:50	1:51–1:270	>1:270	P*
Undecided (N-1,926)	44 (2.3 %)	896 (46.5 %)	986 (51.2 %)	0.26
Yes (N-434)	17 (3.9 %)	204 (47.0 %)	213 (49.1 %)	
No (N-283)	9 (2.6 %)	140 (49.5 %)	134 (47.3 %)	
		U vs. Y	0.14	
		U vs. N	0.37	
		Y vs. N	0.75	

*Fishers exact test

**Table 6:** Screen risk for T21 and decision to pursue stepwise sequential screening

Group	≥1:50	1:51–1:270	>1:270	P* 0.63
Undecided (N-1,926)	26 (1.3 %)	86 (4.5 %)	1,814 (94.2 %)	
Yes				
(N-434)	7 (1.6 %)	21 (4.8 %)	406 (93.5 %)	
No				
(N-283)	7 (2.5 %)	13 (4.6 %)	263 (92.9 %)	
			Uvs. Y	0.79
			U vs. N	0.30
			YvsN.	0.74

*Fishers exact test

The number of patients whose first trimester screen-risk for T21 exceeded their age-risk, and their choice of follow-up, is depicted in Table 7. Overall, 126 patients (4.8 % of the study population) had a first trimester screen-risk for T21 which was greater than their age-risk. Each group had a similar percentage of these patients. Of these, 73(57.9 %) women elected not to have follow-up Stepwise Sequential screening or diagnostic testing, 24(19.8 %) had Stepwise Sequential screening only and 29(23.0 %) had invasive testing via CVS (*N* = 11) or amniocentesis (*N* = 18). The karyotype was normal in 27/29. There was one case of 47, XY, +21 and one apparently balanced Robertsonian translocation between chromosomes 13 and 14. Women who originally declined Stepwise Sequential screening were significantly less likely to pursue invasive testing than women in the other two groups.

**Table 7:** Screen Risk for T21 Greater than Age Risk and Choice of Follow-up

Group	SR > AR	*p*	No Further Testing	SS Screen Only	Invasive Testing	p*
Undecided (n-1,926)	91 (4.7 %)	0.98	52 (57.9 %)	15 (19.0 %)	24 (26.4 %)	
Yes (N-434)	21 (4.8 %)		8 (38.1 %)	9 (42.9 %)	4 (19.0 %)	
No (N-283)	14 (4.6 %)		13 (92.9 %)	0	1 (7.1 %)	
∑ 2,643	126 (4.8 %)		73 (57.9 %)	24 (19.0 %)	29 (23.0 %)	
	U vs. Y	0.92			U vs. Y	0.05
	U vs. N	0.87			U vs N	0.04
	Y vs. N	0.99			Y vs. N	0.002

*Fisher's exact test

A total of 91(3.4 % of the study population) women had invasive testing by amniocentesis (*N* = 76) or CVS (*N* = 15) during the study period. In 59(64.8 %), the age-risk for T21 was greater than the screen-risk. The karyotype was normal in 87/91(95.6 %). There were three cases of T21 and the aforementioned translocation. In two of the three T21 cases, the maternal age risk was greater than the screen risk (The age risks were 1:43 and 1:235 versus screen risks of 1:96 and 1:246, respectively).

The number of invasive procedures performed prior to the availability of first trimester screening, following initiation of first trimester screening and following the availability of Stepwise Sequential screening is indicated in Table 8. The indications for invasive testing were varied and included AMA, abnormal ultrasound findings, abnormal screening, and family history of aneuploidy. All patients in the study population had screening prior to their invasive testing. The number of amniocenteses in our center declined by 51.4 % following the availability of first trimester screening with a further 17.4 % reduction upon initiation of Stepwise Sequential screening. The reduction was significant between all time periods. The number of CVS procedures declined by 19.2 % following availability of first trimester screening. There was a significant reduction in the number of CVS procedures from pre-first trimester screening and its utilization. However, there was no significant difference in the number of CVS procedures between availability of first trimester screening and Stepwise Sequential screening.

**Table 8:** Number of Invasive Procedures Before and After First Trimester and Stepwise Sequential Screening Availability

Procedure	Pre-FTS	FTS^a^ only	SS^b^ Avail	p, pre-FTS vs FTS only	p, FTS only vs. SS avail
Amniocentesis	1,632	793	655	*p* < 0.0001	*p* < 0.0001
CVS	494	399	371	*P* < 0.001	*P* = 0.31

^a^First trimester screening

^b^Sequential screening

## Discussion

In our population, only 20 % of women having first trimester screening, elected to pursue Stepwise Sequential screening with 3.4 % undergoing invasive testing. Proximity to the medical center did not affect the likelihood of our population to return for Stepwise Sequential screening.

Women of lower education level and Black or Hispanic ancestry were least likely to desire further screening or testing following their first trimester screen results. The reason for this disparity is uncertain. It is possible this represents lower health literacy in these groups with less of an appreciation or even misinterpretation of the consequences and benefits of Stepwise Sequential screening. The amount of information to be collected and conveyed at the initial prenatal visit, in addition to decisions regarding aneuploidy screening, can be overwhelming even for relatively medically sophisticated women. Perhaps women of lower education and minorities should be offered pre-conception counseling so that they have more time to consider the benefits and risks of genetic screening and testing. It may also be that women in these groups are more conservative in their approach to prenatal screening overall, and consider themselves less likely to act on the basis of a “positive” result. Additionally, it is possible that their perception and tolerance of risk may be different than women in other groups due to differences in their own frame of reference.

How results are reported, and its potential impact on how a woman interprets this information was a focus of this work. A woman whose screen-risk of T21 is 1:310 will be considered to have a “negative” result and may well be reassured even if this is higher than her age-related risk. By the same token, a woman with a screen-risk of 1:260 will have a result reported as “positive” or elevated risk even if it is a significant reduction compared to her age-related risk. The difference between 0.32 % (1:310) and 0.38 % (1:260) is not great but the implications for a woman’s attitude, anxiety and subsequent plans for screening and testing may be significant. For many patients, interpretation of risk is influenced more by emotions than by facts (Paling 2003). The manner in which risk information is presented or “framed” assumes special importance when it involves communication of genetic testing results. Framing can significantly impact health choices (Edwards et al. 2001; O’Doherty and Suthers 2007). Negative framing, in this case the designation of a test result as indicating elevated risk, may have a greater impact on the perceived need for follow-up testing, than the numerical description of risk, which is more understandable to many patients. This is consistent with evidence that numeric communication of risk is often preferred by individuals when making major medical decisions (Lipkus 2007). The intent of our approach was that the women’s perceptions of their risks would be based on their screen-risk for T21 relative to their age-risk, and not necessarily relative to the risk of a 35-year old woman.

With respect to implications for genetic counselors, an appreciation of the potential impact of framing as it relates to prenatal screening results is appropriate. The way information is presented can significantly affect decisions made. It is important that the magnitude of the increase in risk, whether increased or decreased, be presented as simply and effectively as possible. Classifying a result as “positive” because the T21 risk exceeds that of a 35-year old, may be less useful and more frightening than expressing the screen-risk relative to a woman’s age-risk. Verbal communication of risk is commonly used in other areas of life (it is “likely” to rain, a certain condition is “common”, etc.). The limitations of this approach when applied to medical screening is that it lacks precision due to variability in interpretation of what represents increased risk. Describing a result as “negative” has the effect of providing maximum reassurance, even is cases where the screen related-risk is similar to a woman’s age-related risk. Conversely, informing a woman her result is “positive” is often associated with great stress, which may be ameliorated when the numeric risk is placed in the context of a woman’s age-related risk. Because of the imprecision associated with terms like positive, negative, high-risk, and low-risk, it is our recommendation that when screen results are discussed, numeric risks should be stressed over verbal description of risks. It is possible results presented in this manner facilitate informed choice. Additionally, an absolute number as expressed by the screen-risk provides the patient with an estimate not only of the risk for T21, but also the chance the pregnancy is not at risk. A screen-risk of 1:1,000 or less, not an unusual result, predicts not only a 1:1,000 chance for T21, but roughly a 999:1,000 a fetus does not have T21. The numerical expression of risk allows not only a precise estimate of the risk of a problem, but also the likelihood of normalcy or absence of a condition. In this way, risks can be presented in a balanced manner, not merely focusing on whether a result is positive or negative.

Unlike other forms of medical screening, which are designed to predict or prevent disease, prenatal screening provides women with information allowing them to make informed choices regarding their prenatal care as well as continuation or interruption of their pregnancy. In our approach, a “positive” test can be defined practically as one which prompts further screening and/or diagnostic testing. Similarly, a “negative” test provides sufficient reassurance to obviate further testing or to allow diagnostic testing via amniocentesis rather than CVS. As demonstrated by the First and Second Trimester Evaluation of Risk (FASTER) Trial, detection rates and screen positive rates are modestly improved with the addition of maternal serum Quad screening (Ball, et al. 2007). However, the majority of our population did not require or desire this information to make informed choices regarding their pregnancy. While this may reflect the generally conservative nature of our population, it may also indicate the information, when presented as described, facilitates women’s understanding of the change in absolute risk for fetal trisomy relative to their baseline, age-related risk. This is consistent with evidence that responses to risk often depend on that to which the calculated risk is being compared (Johnson 2004). It also reflects the contextualized nature of prenatal risk assessment and the observation that objective risk and perceived risk are often dissimilar (Marteau 1999). The influence of other factors, including religious, economic, and the perceived effects of a child with aneuploidy on the existing family structure also play an important role in the patient interpretation of and response to screening results (Garcia et al. 2012).

The availability of first trimester screening and Stepwise Sequential screening were each associated with a significant decline in the number of amniocenteses performed at our institution. The smaller decrease between first trimester and Stepwise Sequential screening can be at least partially attributed to the fact only 20 % of our population elected sequential screening. First trimester screening was also associated with a significant reduction in the number of CVS procedures performed.

The option of Stepwise Sequential screening did not further reduce the number of CVS’s. This is not unexpected as most pregnancies at increased risk for fetal aneuploidy are identified earlier with the option for first trimester diagnostic testing, obviating the need for mid-trimester testing. This also reflects some cases where the screen risk was lower than the maternal age risk but the reduction was not adequately reassuring, resulting in the decision to proceed with earlier diagnostic testing. With respect to the utilization of invasive procedures, our findings are similar to those described by others. Wray, et al. reported that availability of first trimester screening coincided with more women of advanced maternal age (AMA) referred for early genetic counseling. In 2001, 68 women were seen for early genetic counseling versus 172 in 2003. The rates of invasive testing dropped significantly from pre- to post-first trimester screening availability (71 % vs 26 %, *p* < 0.01). The types of invasive testing were not specified (Wray et al. 2005). Benn, et al. reviewed a series of nearly 2000 amniocentesis and CVS samples from 1991 to 2002. They reported a 50 % reduction in the number of invasive procedures over this time. This was attributed to advances in maternal serum screening and mid-trimester sonography. However, this was before the widespread application of first trimester screening and it was not stipulated whether nuchal translucency or first trimester serum markers were included in this analysis. Additionally, CVS specimens comprised only 4.3 % of their samples (Benn, et al. 2004).

It is felt that first trimester screening is the major reason for the decrease in invasive procedures. In our experience, prenatal care providers have embraced this technology and routinely offer this option even to at-risk women, ahead of invasive testing. It is possible this predisposes women to have confidence in first trimester screening sufficient to obviate the need for absolute reassurance via invasive testing with its attendant risks. It is also possible the risks of invasive testing are exaggerated in many womens’ minds. There may also be an element of “quit while ahead”. If you have a result you like, why seek additional information which may detract from your peace of mind? In our opinion, receiving a very low risk early in pregnancy gives many women the reassurance that their pregnancies are “normal”. It may be that this makes it easier for some to believe that further testing is unnecessary. Similar reassurance can be provided by maternal serum Quad screening, but not until later in the pregnancy.

The main limitation of our study is that it reflects a local population whose attitudes regarding screening and testing and thresholds for pursuing follow-up may not be reflective of women in other parts of the country. Additionally, while most cases of T21 and T18 are reliably diagnosed postnatally, the majority of women did not have cytogenetic testing and postnatal follow-up was unavailable for 64 patients. Therefore, other undetected cases of aneuploidy cannot be excluded. Other limitations include the fact that there is no control population of women who did not receive results reported as “positive” or “negative”. We are unable to directly compare our approach with other methods of risk communication. It is also plausible that both health care providers and patients are used to considering medical test results as normal or abnormal, and may be less familiar with the notion of comparative quantitative risk assessment. Lastly, while noninvasive prenatal testing with cell free fetal DNA is presently limited, the anticipated expanded application of such technology will likely limit the utilization of current screening paradigms. Therefore, other risk communication and counseling strategies will ultimately need to be developed to suit different circumstances.

## Conclusion

In summary, communication of risk for aneuploidy is especially important and challenging in the prenatal setting. Following the performance of first trimester screening, 80 % of our study population ultimately declined follow-up testing, including nearly one-half of those who initially planned to pursue Stepwise Sequential screening. The policy of providing patients with their results by specifying their age-related risk, their revised screen-related risk and further options, without classifying results as “positive” or “negative”, seemed to communicate risk in a manner which was understandable and clinically useful, with minimal bias. This also resulted in far fewer Stepwise Sequential screens being performed than would be predicted using most current sequential screening paradigms. The introduction and increasing utilization of first trimester combined screening has been accompanied by a steady decrease in the number of invasive diagnostic procedures, which is more pronounced for amniocentesis than CVS.
